# Multiparametric Magnetic Resonance Imaging for Active Surveillance of Prostate Cancer

**DOI:** 10.4274/balkanmedj.2017.0708

**Published:** 2017-09-29

**Authors:** Julie Y. An, Abhinav Sidana, Peter L. Choyke, Bradford J. Wood, Peter A. Pinto, İsmail Barış Türkbey

**Affiliations:** 1 Center for Interventional Oncology, NIH Clinical Center and National Cancer Institute, National Institutes of Health, Maryland, USA; 2 Urologic Oncology Branch, National Cancer Institute, National Institutes of Health, Maryland, USA; 3 Molecular Imaging Program, National Cancer Institute, National Institutes of Health, Maryland, USA

**Keywords:** prostate cancer, Oncology, imaging, prostate specific antigen, multiparametric magnetic resonance imaging, active surveillance, PI-RADS

## Abstract

Active surveillance has gained popularity as an acceptable management option for men with low-risk prostate cancer. Successful utilization of this strategy can delay or prevent unnecessary interventions - thereby reducing morbidity associated with overtreatment. The usefulness of active surveillance primarily depends on correct identification of patients with low-risk disease. However, current population-wide algorithms and tools do not adequately exclude high-risk disease, thereby limiting the confidence of clinicians and patients to go on active surveillance. Novel imaging tools such as mpMRI provide information about the size and location of potential cancers enabling more informed treatment decisions. The term “multiparametric” in prostate mpMRI refers to the summation of several MRI series into one examination whose initial goal is to identify potential clinically-significant lesions suitable for targeted biopsy. The main advantages of MRI are its superior anatomic resolution and the lack of ionizing radiation. Recently, the Prostate Imaging-Reporting and Data System has been instituted as an international standard for unifying mpMRI results. The imaging sequences in mpMRI defined by Prostate Imaging Reporting and Data System version 2 includes: T2-weighted MRI, diffusion-weighted MRI, derived apparent-diffusion coefficient from diffusion-weighted MRI, and dynamic contrast-enhanced MRI. The use of mpMRI prior to starting active surveillance could prevent those with missed, high-grade lesions from going on active surveillance, and reassure those with minimal disease who may be hesitant to take part in active surveillance. Although larger validation studies are still necessary, preliminary results suggest mpMRI has a role in selecting patients for active surveillance. Less certain is the role of mpMRI in monitoring patients on active surveillance, as data on this will take a long time to mature. The biggest obstacles to routine use of prostate MRI are quality control, cost, reproducibility, and access. Nevertheless, there is great a potential for mpMRI to improve outcomes and quality of treatment. The major roles of MRI will continue to expand and its emerging use in standard of care approaches becomes more clearly defined and supported by increasing levels of data.

Prostate cancer (PCa) is the most common non-cutaneous cancer among American men, and the second leading cause of cancer death (1). Despite high prevalence of disease, most PCa tumors are indolent and unlikely to progress into clinical significance. The current direction of low-risk PCa management is towards active surveillance (AS), which is a way to monitor localized PCa, rather than treating it right away. Magnetic resonance imaging (MRI) technology has gained adoption in recent years for its superior ability to visualize prostate lesions. Patients can be assessed for their candidacy in AS using MRI, and be biopsied with the more accurate targeted approach. The purpose of this review article is to provide a brief introduction to the current status of PCa management using MRI, and to critically evaluate the growing role this technology has in AS of men with low-risk PCa.

## THE PROSTATE CANCER SCREENING DEBATE

Screening with the prostate specific antigen (PSA) blood test and digital rectal exam (DRE) led to random biopsies that dramatically increased the number of PCa diagnosis beginning in the late 1980s and early 1990s (2,3). It was argued that the increased detection of low-risk cancer diagnosis and subsequent overtreatment was not beneficial to patients and resulted in a net harm from screening (4,5,6). The Prostate, Lung, Colorectal, and Ovarian (PLCO) Cancer Screening Trial, a prominent trial conducted in the United States, found no survival advantage in the men randomized to the PSA screening arm relative to their control. This finding was in part responsible for the letter grade of “D” assigned by the United States Preventive Services Task Force (USPSTF) in 2012 resulting in a decline in PSA screening of 18% across all races and groups (7). Those who support screening initiatives have been highly critical of the PLCO’s “intent to treat” study design, which was a major pillar of the conclusion. Concerns over crossover and control group contamination with PSA put the reliability of this study in question. Shoag et al. (8) noted that over 90% of the men in the “usual care” non-screening arm actually underwent PSA testing outside of the trial negating any possible interpretation of the study. In light of recent warnings over the rise of metastatic PCa and the recognition that the data on which the decision was based was highly flawed, the USPSTF recently revised its recommendation to a letter grade of “C”, to encourage a discussion between the patient and physician regarding PSA screening (9).

## ACTIVE SURVEILLANCE

There are undoubtedly morbidities associated with PCa screening but it is undeniable that during the PSA era beginning in the early 1990s, the mortality rate of PCa has steadily declined. While many explanations have been postulated, a perfectly valid explanation is that screening reduces PCa mortality. However, it is also undeniable that during the PSA era, many men were over-treated. It is increasingly recognized that low-risk PCas, consisting of low volume Gleason 3+3 cancers, do not require treatment and can be safely watched. Integrating this kind of risk stratification into treatment decisions changes the calculus behind screening. During the early years of screening, many men elected for definitive radical prostatectomy (RP) or radiotherapy regardless of PCa risk score. To limit harms associated with overtreatment, new strategies for management based on individual and disease factors have been explored. AS is the most well-known approach to low risk cancers. The main objective of AS is to prevent overtreatment of men with low-risk PCa that is unlikely to progress. AS involves monitoring patients for progression and to offer treatment within a window of curability. Details in AS eligibility and follow-up protocols vary among institutions (Table 1), but are generally comprised of periodic PSA, DRE, and biopsy to monitor for disease progression. AS has quickly gained popularity as an acceptable option for low and low-intermediate-risk PCa, and is effective in lowering the burden of over diagnosis and overtreatment addressing much of the initial concerns regarding PCa screening. The exact role of MRI within AS approaches remains to be fully validated and defined, but the strengths of MRI match some of the clinical requirements for AS populations.

Surveillance programs allow for an adequate window of curability, without compromising disease specific mortality (10). This strategy was historically underutilized in the US, with only 6.2% of AS-eligible patients being monitored under AS protocols from 2001-2010 (11). Recent shifts in practice and policy have increased the number of patients opting for AS to 40% - 49% (12,13,14). Two prominent series with greater than 15-years follow-up contributed to AS’s wide acceptance. Cohorts at Johns Hopkins and the University of Toronto have shown disease-specific survival of 99.9% and 94.3%, respectively (10,15). Similar trends were reported in Australia and Sweden (16,17,18). The rate of aggressive treatment for low-risk PCa has declined reflecting greater adoption of conservative measures. Louis et al. (16) reported a significant decrease in the number of RPs performed on low-risk PCa from 2007 to 2012. This strategy is efficacious when the appropriate patients are selected and followed closely. However, full compliance does not always occur due to a myriad of factors.

The usefulness of AS depends primarily on correct identification of those with low-risk disease. However, our current methods of diagnosing PCa do not adequately exclude high-risk disease-thereby limiting the confidence of clinicians and patients to go on AS. The diagnosis of PCa is unique in that pathologic tissue is most commonly sampled “blindly”, as opposed to other cancers that are diagnosed with image guidance. Although transrectal ultrasound (TRUS) is used during prostate biopsy to assist in the guidance of needle placement, it is limited in its ability to visualize the tumor. Imaging modalities used for the diagnosis of other cancers also allow for visualization of disease extent, but in PCa, clinicians must estimate extent using risk factors, PSA, DRE, and systematic untargeted biopsy results. Imaging with multiparametric MRI (mpMRI) provides much-needed information about the size and location of potential tumors, especially intermediate-high risk tumors, enabling more accurate diagnosis and steering such patients away from AS and toward active treatment. The hidden higher-Gleason anterior lesion may be seen with MRI, and is often undersampled and undiagnosed by blind TRUS biopsy. This provides a good example of the potential role of MRI in this AS population.

## PROSTATE MULTIPARAMETRIC MRI

mpMRI was developed in response to the critical need for better imaging of the prostate. The strength of mpMRI lies in its superior soft tissue resolution with anatomic zonal delineation (19), making it particularly useful for distinguishing indolent from aggressive disease. Refinement of mpMRI has also allowed for more accurate biopsies. Although this technology was once limited to large tertiary academic centers, it’s use has permeated deeply into the community in the USA in recent years.

The term “multiparametric” refers to a combination of MR series that includes T2 weighted imaging, diffusion weighted imaging and dynamic enhanced imaging. The development of this technique and has been the key to its success in prostate imaging. The main advantages of MRI are its superior anatomic and contrast resolution, lack of ionizing radiation and multi-planar capabilities. In prior years, image acquisition, protocol, interpretation and reporting varied greatly. In early 2015, the American College of Radiology in conjunction with the European Society of Urogenital Radiology released the Prostate Imaging-Reporting and Data System (PI-RADS) version 2 in order to standardized guidelines and mitigate inconsistencies (20).

The imaging sequences in mpMRI defined by PI-RADS version 2 include: T2-weighted (T2W MRI), diffusion-weighted (DW MRI), apparent-diffusion coefficient (ADC maps) derived from DW MRI, and dynamic contrast-enhanced (DCE MRI) imaging (20). The main anatomic sequence is the standard T2W MRI (20). Here, PCa is typically low in signal intensity due to reduced water content, with high cellular density and desmoplastic reaction (19). The higher resolution of T2W MRI allows assessment of extra-prostatic extension involving the peri-prostatic fat, seminal vesicles, neurovascular bundle, and adjacent organs (21). However, T2W MRI alone is not sufficient for detection and localization of PCa since many inflammatory and hyperplastic changes appear similar to PCa.

The addition of DW MRI sequences improves MRI’s sensitivity and specificity for PCa. DW MRI is comprised a series of lower b values (typically 0-1000 sec/mm^2^), the high b value DW MRI (typically >1400 sec/mm^2^) and ADC maps (22). It is the dominant sequence used to categorize lesions in the peripheral zone of the prostate. The term “diffusion” refers to the dependence of this sequence on the motion of water molecules within tissue, the greater the diffusion of water, the lower the signal on raw images and the higher the ADC value (20). Tumors exhibit crowding of cells relative to normal tissue and therefore show restricted water movement (20). A critical part of the DWI suite of sequences is high b-value imaging in which the b-value ranges from 1400-2000 sec/mm^2^. This is employed to obtain superior suppression of benign tissue while retaining signal in tumors (23). While DWI MRI is very useful in the peripheral zone, it is less useful in the transition zone because benign prostatic hyperplasia (BPH) nodules can exhibit properties similar to PCa (24,25). A major issue with DWI is that it is prone to distortion and warping artifacts due to even small amounts of rectal gas or body motion.

DCE MRI is the third sequence in the prostate mpMRI protocol. DCE is used to confirm suspicion of lesions seen on other sequences, and to direct radiologists’ attention to areas that may have been overlooked. PCa, DCE MRI is useful primarily for its ability to detect areas with increased vascularity related with tumor angiogenesis. However, not all tumors have high vascularity, so DCE MRI cannot be used alone. Moreover, other pathologies also exhibit increase enhancement on DCE MRI such as infection, inflammation and BPH. This sequence is performed with fast T1 weighted imaging after the rapid injection of gadolinium contrast media. PI-RADS version 2 advises high temporal resolution of less than 10 seconds per 3D acquisition. “Dynamic” visual assessment is used to look for early enhancement and rapid washout correlating with suspicious tumors. This replaces more complex analysis of kinetic enhancement curves, and parametric maps that were once used to analyze DCE MRI. However, the diagnostic yield has not improved with the addition of these kinetic models of analysis (over standard DWI/ADC) and they have largely been abandoned.

MR spectroscopy imaging, a technique that helps identify abnormalities in specific tissue metabolites, is sometimes included in prostate mpMRI protocols. High levels of choline relative to citrate within a region of interest are characteristics of PCa. However, spectroscopy is not included in the PI-RADS version 2 protocol due to difficulties in standardizing acquisition and difficulties in analyzing the data. Moreover, it takes almost 15 minutes to acquire, is susceptible to many artifacts and in general, has not proven its worth over time (20).

After appropriate assessment of each sequence on mpMRI, each lesion is assigned a PI-RADS score ranging from 1 to 5. PI-RADS scores reflect the likelihood of harboring clinically significant PCa with “1” having the lowest, and “5” having the highest suspicion. Differential diagnosis for lesions seen on mpMRI include bleeding after prostate biopsy, BPH nodules, chronic or acute inflammation caused by prostatitis, or abscess (20). This PI-RADS system has been validated and correlates with the rate of clinically significant cancer PCa (26,27,28). However the importance of robust MRI acquisition, experienced radiologists, and structured reporting cannot be underestimated, and is requisite to value of this approach. Reliability of clinical information depends greatly on these factors, so consistent use of standardized protocols is essential.

## EVOLVING ROLE OF MULTIPARAMETRIC MRI IN ACTIVE SURVEILLANCE

**FIG. 1.**
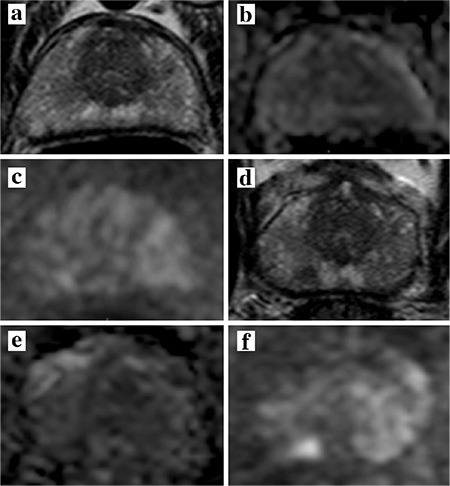
Fourty six-year old man with a serum PSA= 6.79 ng/mL with Gleason 3+3 prostate cancer diagnosis. Baseline mpMRI consisted of T2W MRI (a) ADC map (b) b1500 DW MRI (c) shows no lesion at the level of right apical portion of the prostate. Two year follow up axial T2W MRI (d) shows a lesion in the right apical peripheral zone, which is also positive on ADC map (e) and b1500 DW MRI (f) (serum PSA at 2 year follow up= 9.25 ng/mL). TRUS/MRI fusion biopsy revealed Gleason 4+3 with this lesion and patient became a radical prostatectomy candidate.

**FIG. 2.**
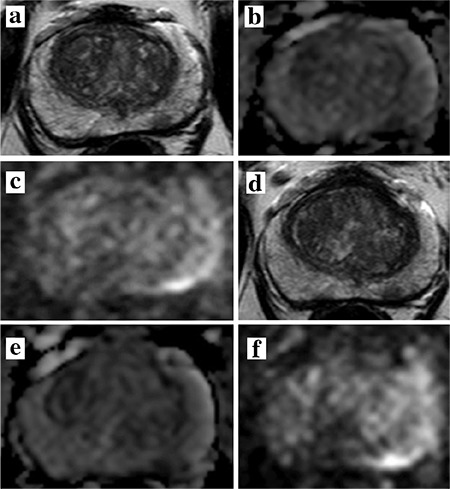
Sixty-year old man with a serum PSA= 4.58 ng/mL. Baseline mpMRI consisted of T2W MRI (a) ADC map (b) b1500 DW MRI (c) shows a focal lesion in the left mid peripheral zone. TRUS/MRI fusion guided biopsy revealed Gleason 3+3 prostate cancer within the lesion. One year follow up axial T2W MRI (d) ADC map (e) and b1500 DW MRI (f) shows no significant change within the lesion (serum PSA at 1 year follow up= 5.10 ng/mL). TRUS/MRI fusion biopsy revealed Gleason 3+3 with this lesion and patient continues to remain on active surveillance.

AS is an increasingly important option following a PCa diagnosis. Suitable candidates include patients with low-grade cancers with lower PSA values and small volume. However, as many as 60% of AS patients come off of AS after 10 years (29). This high rate of “progression to treatment” is part real, and is part due to missing clinically significant cancer at the initiation of AS (that are found subsequently). mpMRI has been documented to be a useful imaging technique in detecting localized PCa and estimating tumor volume even in challenging locations of the prostate such as the anterior transition zone, central zone and distal apex (30). There are several scenarios in which mpMRI is particularly useful in patients initially considered eligible for AS. The most important role is in upgrading or ruling out more significant cancer. Other utilities include: determining the size and extent of tumor after diagnosis, assessing for growth, localizing tumor in patients with persistently rising PSA despite negative biopsy, and follow-up of patients after therapy. The role of mpMRI in AS is reviewed here. Specific clinical case examples of the use of mpMRI in AS are provided in Figure 1 and 2.

## EVOLVING ROLE: EVALUATING ACTIVE SURVEILLANCE CANDIDACY

During the evaluation of AS candidacy, patients are stratified into risk categories based on clinical and pathologic results. When considering AS there is great concern over the possibility of under-sampling and under-estimation of the extent of disease. If patients harboring high-risk disease are wrongly placed in a lower risk category, they risk having preventable disease progression while under AS. Traditional tools used to determine if AS is appropriate (PSA, DRE, and TRUS guided biopsy) are only surrogates for assessing disease burden, and may not accurately determine extent nor predict progression (31,32). PSA fluctuates greatly with activity (33), the utility of DRE is subjective and location dependent (34).

The “blind” 12-core TRUS biopsy on which much of the clinical decision-making is based, may not reflect the actual disease burden if the lesion is located in a challenging position to biopsy such as the anterior transition zone or distal apex (30). Numerous studies have shown upgrading of tumors after initial biopsy, providing evidence that this random biopsy technique is not sufficient to rule out significant cancer (35,36,37,38). In a study from Johns Hopkins, 557 patients on AS for “very low” and 251 “low” risk cancers were initially placed on AS. After repeat biopsy at 2 year follow-up, 35% of these men had upgrading in their Gleason classification (39). It is unlikely the high rate of upgrading was entirely due to time related disease progression. It is more likely, however, that there was under-sampling at the time of initial biopsy. If the latter is the case, many of these patients may have been wrongly placed on AS to begin with. In a separate prospective study of 582 patients with clinical suspicion of PCa, standard 12-core biopsy as well as MR/US fusion guided biopsy were performed during the same procedural session (40). 32% of men had higher Gleason grade tumors detected using the targeted biopsy vs. standard biopsy technique (40). If AS candidacy was evaluated using only the 12-core biopsy, approximately 1/3 of patients would have been incorrectly assigned initially by one 12-core biopsy to AS, and thereby received insufficient treatment.

In a prospective cohort of 45 patients from the National Institutes of Health, suspicious findings on 3T mpMRI were correlated with histology from whole mount prostatectomy specimens (41). mpMRI was able to identify clinically significant (Gleason ≥7) cancers with a PPV of 98% overall, 98% in the peripheral zone, and 100% in the central gland (41). There was also improved sensitivity for detection of larger lesions (≥5 mm), and lesions with higher Gleason grade (Gleason ≥7) (41). This study demonstrated the predictive ability of mpMRI to identify higher-grade cancers even while it missed low grade cancers. A negative or minimally abnormal mpMRI therefores provides patients and caretakers with the assurance to confidently proceed with AS.

Most of the benefit to mpMRI comes from detecting anterior tumors, an area where standard biopsy is lacking (42,43). A cohort of 176 patients with at least one previously negative biopsy and persistently elevated PSA cancer underwent MRI/US fusion guided biopsy. Two hundred seventy seven targets visualized on MRI were targeted, and 202 (73%) of those targets were identified as cancer. Of the cancerous lesions, 141 (70%, 95% CI 63-78%) originated from the anterior zone (42). Anterior lesions are difficult to reach on standard TRUS biopsy (42,44). In a retrospective study, Shinmoto et al. (43) evaluated 87 patients who underwent 3T mpMRI prior to RP for anterior lesions. Radiologists interpreted two protocols of prostate MRI, one of T2W MRI alone and one of T2W MRI with an ADC map. ROC analysis demonstrated that the AUC increased from 0.75 to 0.88 for the identification of lesions with the addition of ADC maps, improving both sensitivity and specificity (43).

Advancements in mpMRI have coincided and enabled the development of MRI-US fusion guided biopsies. The ability of mpMRI to detect subtle differences in soft tissue makes it a powerful tool for guided biopsy. The targeted biopsy approach has been repeatedly demonstrated improved detection of high grade tumors, while avoiding insignificant tumors (21,38). In a prospective, single institution study of 1003 patients with MR visible lesions, Siddiqui et al. (38) showed a 37.5% higher diagnostic rate for detection of clinically significant cancers using MR/US fusion biopsies (37.5%) versus standard 12 core biopsies (26.5%).

The UK National Institutes of Health and Care Excellence has already recommended mpMRI as a part of their AS initiation protocol (45). Increasingly, mpMRI is employed before committing a patient to AS and this is likely to be codified in practice guidelines in the near future.

Patient hesitancy to “sit on” a cancer diagnosis is a major deterrent to the use of AS. In one large retrospective analysis of 24.450 patients with low risk PCa suitable for AS, over half (55%) selected definitive treatment over AS (46). Kelly et al. (47) found 27% of patients initially on AS opted for definitive treatment within 2.9 years of follow-up. Although practice patterns have shifted over the last decade towards more conservative treatments, the role of mpMRI in maintaining men on AS has not been explored. mpMRI could have great value for apprehensive patients if it can provide reassurance prior to entering the program.

## EVOLVING ROLE: MONITORING FOR PROGRESSION

MRI may also help identify patients for AS as well as monitor patients on AS (Figure 1, 2) A recent study from Felker et al. (48) examined whether increased suspicion score on serial mpMRI predicts pathologic progression on repeat biopsy. The mean interval time between baseline and follow-up imaging and biopsy in this group was 28.3 months (range 11-43 months). Serial mpMRI along with initial biopsy results and PSA density were predictive of pathologic progression with an AUC of 0.91 compared to 0.87 for biopsy and PSA results alone (p=0.044) (48). This study suggests serial mpMRI has the potential to predict upgrading in men on AS. Those with stable imaging findings can be reassured that their disease has not progressed, preventing early termination of AS for unnecessary therapy and potentially avoiding additional biopsies. Although financial and accessibility factors limit the dissemination of serial mpMRI protocols at this time, this study gives a proof of concept for future development.

It must be emphasized that mpMRI is imperfect for diagnosing PCa and has not been fully tested over the long term in AS. Changes in size and extension into surrounding areas generally infer progression, and should prompt re-biopsy and possible discontinuation of AS. However, there is currently no consensus or criteria on what metrics defines radiologic progression and how predictive this is of real pathology-proven progression. A prospective single-institution study led by Habibian et al. (49) followed patients on an AS protocol who were monitored with annual mpMRI in place of serial biopsies. The objective of the study was to report imaging characteristics that suggest tumor upgrading and disease progression. Of the 114 patients followed with at least one follow-up, 14 patients had mpMRI concerning for progression. Of these, 3 (21.4%) patients had enlargement of previously identified lesions, 2 (14.3%) patients were identified with new lesions, and 9 (64.3%) patients showed new extracapsular extension. Biopsy in these 14 patients revealed progression in 43% of these patients. This study was limited by sample size and PCa’s inherent low rate of disease progression. Future research would assist in defining what imaging characteristics are strongly indicative of progression on AS.

Although AS stands as a reasonable approach for low-risk PCa, there are significant challenges in patient compliance. The National Comprehensive Cancer Network guidelines recommend patients on AS to undergo repeat biopsy every 12 months and repeat PSAs every 6 months (50). Biopsies are uncomfortable and anxiety provoking for patients, and associated with potentially dangerous complications such as hematuria, rectal bleeding, and infection. A prospective registry comprised of AS patients from 42 independent practices, showed a staggering dropout rate of 69.4% (12). Out of the entire cohort, 53.6% of the patients had dropped out due to noncompliance particularly centered on the repeat biopsy requirement (12). Additional studies have also reported similar rates of noncompliance (51). In a retrospective analysis of 45 AS patients from the Kansas City Veterans Affair database (100%) of these patients complied with the repeat PSA requirement, but only 34 (53.3%) patients complied with the mandated repeat biopsy requirement. With significant concerns of compliance and discomfort, there is a clinical need for improved methods of monitoring patients on AS. MRI may be able to mitigate some anxiety in patients if it can be used in place of repeat biopsy or as a way to defer biopsies. There is currently no consensus on the appropriate length of time between repeat biopsy for patients on AS and it is institution-dependent (ranging from 12-36 months) (17,31,32,50). Rais-Bahrami et al. (52) aimed to determine the natural history of low-grade Gleason 6 lesions (≤7 mm and ≤5 mm) lesion. After 2 years, they found no significant change in size in either the ≤7 mm or the ≤5 mm groups. These findings suggest surveillance intervals of at least 2 years may be appropriate, as these small lesions with low grade have negligible growth rates (52). When used in a serial fashion, mpMRI may also allow for increased intervals between biopsies (53). Replacing biopsy with non-invasive imaging alternatives would likely result in greater compliance and reduction of procedure-associated complications.

In conclusion, AS has become an acceptable management option for men with low-risk PCa. Successful utilization of this strategy can delay or prevent unnecessary interventions - thereby reducing morbidity associated with overtreatment. The usefulness of AS primarily depends on correct selection of patients with low-risk disease. mpMRI has been effectively utilized for identifying patients with low-risk PCa appropriate for AS in several moderately sized trials. The use of mpMRI could prevent those with, high-grade lesions from going on AS, as well as assure those who may be hesitant about AS. These diagnostic and monitoring protocols are still being optimized, with significant efforts at consensus building and standardization. Few centers have started annual mpMRI for follow-up of men on AS in lieu of biopsy. Although larger validation studies are still necessary, preliminary results are encouraging. Currently the biggest obstacles to routine use of prostate MRI are quality control, standardization of technique and interpretation, cost, and access. Nevertheless, there is great a potential for mpMRI to improve outcomes and appropriate stratification for treatment for men with PCa. MRI will likely play a growing role in standard of care for the PCa patient (54,55).

## Figures and Tables

**Table 1:**
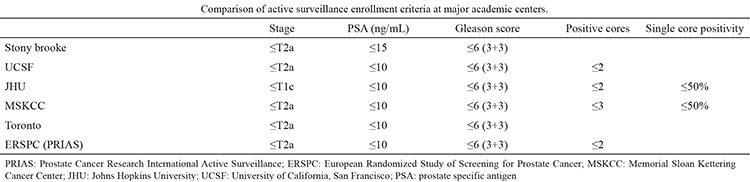
Active surveillance enrollment criteria at major academic centers
